# Structural Evolution of the Protein Kinase–Like Superfamily

**DOI:** 10.1371/journal.pcbi.0010049

**Published:** 2005-10-21

**Authors:** Eric D Scheeff, Philip E Bourne

**Affiliations:** 1 San Diego Supercomputer Center, University of California, San Diego, California, United States of America; 2 Department of Pharmacology, University of California, San Diego, California, United States of America; European Bioinformatics Institute, United Kingdom

## Abstract

The protein kinase family is large and important, but it is only one family in a larger superfamily of homologous kinases that phosphorylate a variety of substrates and play important roles in all three superkingdoms of life. We used a carefully constructed structural alignment of selected kinases as the basis for a study of the structural evolution of the protein kinase–like superfamily. The comparison of structures revealed a “universal core” domain consisting only of regions required for ATP binding and the phosphotransfer reaction. Remarkably, even within the universal core some kinase structures display notable changes, while still retaining essential activity. Hence, the protein kinase–like superfamily has undergone substantial structural and sequence revision over long evolutionary timescales. We constructed a phylogenetic tree for the superfamily using a novel approach that allowed for the combination of sequence and structure information into a unified quantitative analysis. When considered against the backdrop of species distribution and other metrics, our tree provides a compelling scenario for the development of the various kinase families from a shared common ancestor. We propose that most of the so-called “atypical kinases” are not intermittently derived from protein kinases, but rather diverged early in evolution to form a distinct phyletic group. Within the atypical kinases, the aminoglycoside and choline kinase families appear to share the closest relationship. These two families in turn appear to be the most closely related to the protein kinase family. In addition, our analysis suggests that the actin-fragmin kinase, an atypical protein kinase, is more closely related to the phosphoinositide-3 kinase family than to the protein kinase family. The two most divergent families, α-kinases and phosphatidylinositol phosphate kinases (PIPKs), appear to have distinct evolutionary histories. While the PIPKs probably have an evolutionary relationship with the rest of the kinase superfamily, the relationship appears to be very distant (and perhaps indirect). Conversely, the α-kinases appear to be an exception to the scenario of early divergence for the atypical kinases: they apparently arose relatively recently in eukaryotes. We present possible scenarios for the derivation of the α-kinases from an extant kinase fold.

## Synopsis

Most proteins have distinct three-dimensional structures that determine much of their functional capability. Proteins that are related usually have similar structures, owing to their shared genetic heritage and (often) similar function. Hence, one can speak of “families” of proteins that at one time all shared a common ancestor gene, but have diverged over eons of evolution into distinct forms with similar but altered sequences. In some cases, this sequence divergence can occur to the point that the structures of the proteins actually begin to change, forming “superfamilies” of distantly related proteins. Traditionally, events in protein evolution are investigated through the construction of evolutionary trees based on similarity between protein sequences. However, at the superfamily level sequence similarity weakens to the point that building accurate trees becomes much more problematic. This work attempts to address this problem by integrating structural similarity information into the analysis. Because protein structure changes much more slowly than sequence, structural similarity provides powerful signals about the relationships between proteins. When this new form of tree is considered alongside other evolutionary information, the authors are able to provide a supportable history for much of the evolution of the important protein kinase–like superfamily.

## Introduction

A protein superfamily has been defined as a group of proteins that share structure, sequence, and functional features that strongly suggest they are all derived from the same common ancestor protein [1]. However, because protein sequences are highly degenerate, protein superfamily relationships are often not detectable from sequence information alone [2,3]. Protein superfamily relationships often have become apparent when structures of proteins were solved experimentally, only to reveal surprising structural similarities with known structures (e.g., [4]). Hence, structural information provides the gateway through which superfamily-level relationships may be studied. The Structural Classification Of Proteins (SCOP) database classifies proteins hierarchically, based on a tiered class, fold, superfamily, and family system [1]. The superfamilies within the SCOP database are divided up into distinct families of more closely related proteins. Protein families usually display clear sequence similarity and highly similar structures. Hence the “protein landscape” contains families of closely related proteins that share distant common ancestry with other families, forming superfamilies.

The Ser/Thr and Tyr protein kinases are a family of proteins that act as important arbiters of signal transduction in eukaryotes [5–7], and many prokaryotes [8–11]. With the determination of the first protein kinase structure [12], it became possible to place the distinctive protein kinase catalytic core motif into a structural context. The determination of additional kinase structures enforced the notion that the basic fold of the protein kinase catalytic core was structurally well conserved, and had been reused across long evolutionary timescales in a largely intact form [13].

**Figure 1:**
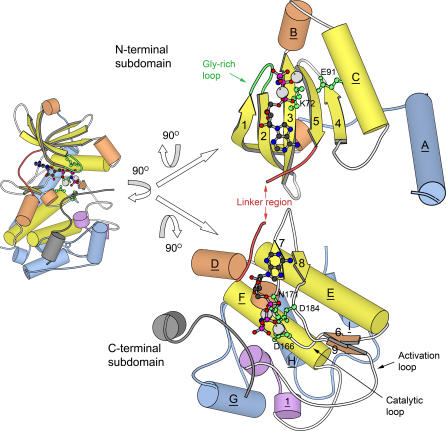
Two Views of the Structure of PKA [70] The structure consists of two subdomains: a small, primarily β-sheet N-terminal subdomain, and a larger, primarily helical C-terminal subdomain. ATP and metal ions are bound in the cleft between the two subdomains. The small left-side view depicts PKA in the “standard” orientation used by the authors when the structure was initially solved [12], and in many subsequent publications. The larger view on the right side depicts PKA in an “open-book” format that makes structural features in the two subdomains easier to compare between families. The open-book view is achieved by rotating the standard view 90° about the vertical axis, then splitting the two subdomains at the linker region and rotating each 90° in opposite directions about the horizontal axis. Helical secondary structures (both α-helices and 3–10 helices) are depicted as cylinders, and β-strands are depicted as arrows. Elements are labeled according to the standard conventions for PKA. Some secondary structure (particularly 3–10 helices) is not labeled in the standard PKA convention, and so is unlabeled here. One structure (Helix 1) was named by us (see text). Underlined labels belong to helical structures; non-underlined labels belong to β-strands. Secondary structure elements are colored according to their conservation status in the overall superfamily as follows: yellow, elements are part of the “universal core” seen in all kinases in the superfamily; orange, elements are present in more than two, but not all, of the kinases in the superfamily; purple, elements seen only in this family, but inserted within in the portion of the chain forming the universal core; blue, elements seen only in this family, and connected to the N- or C-terminal ends of the universal core. A bound pseudosubstrate inhibitor (PKI) is present in the structure [12], and depicted in gray. This inhibitor likely describes the binding location of actual substrates of PKA. The bound ATP molecule is rendered as a ball-and-stick model, while the bound Mg ions are rendered as gray spheres. The ATP and Mg ions are duplicated in mirror image and shown interacting with both the N- and C- terminal subdomains in the open-book rendering. The most critical and highly conserved residues in PKA (and the broader superfamily) are shown as ball-and-stick models in green, and labeled according to the standard PKA numbering scheme. In addition, the glycine-rich loop is also depicted in green, though individual glycine residues are not shown. The loop that forms the linker region between the subdomains is depicted in red. Other loops within the universal core are shown in white, except for loops linking purple regions (which are shown in purple), and loops outside of the universal core (shown in blue). Key loops described extensively in the text are labeled. For increased clarity, residues 300–350 have been removed from the C-terminus of PKA. This loop region is unique to PKA, and would have been colored blue if present in the figure. Molecular renderings in this figure were created with MOLSCRIPT [90].

The protein kinases exert control over their protein targets by covalent modification of a Ser, Thr, or Tyr residue with the γ-phosphate group cleaved from ATP. All of the typical protein kinases (TPKs) share a common catalytic core consisting of a small, mostly β-sheet, N-terminal subdomain and a larger, mostly α-helical, C-terminal subdomain [13] (Figure 1). The ATP binding pocket sits in a cleft between these two subdomains, which can rotate into “open” and “closed” conformations depending on ATP binding and the activation state of the molecule [14–16]. The residues involved in the phosphotransfer reaction sit at the outside edge of the ATP binding region and are highly conserved [13,17].

**Figure 2:**
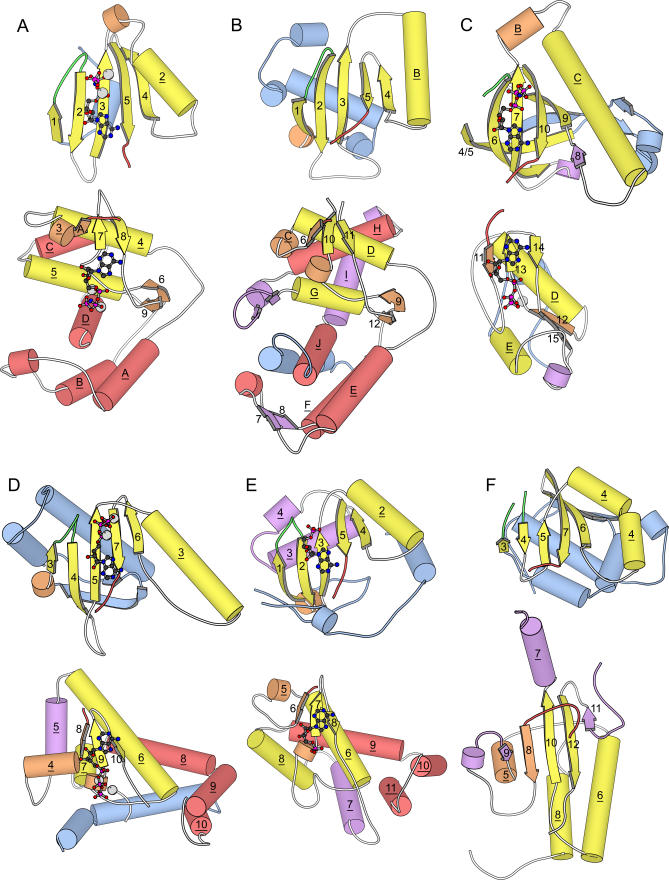
Views of Structural Representatives from Six Families in the Kinase-Like Superfamily Other Than the TPKs Structures are shown in an open-face view, and using the same conventions as used for PKA in Figure 1. ATP and metal ions are shown in mirror image where available in the structure. Similar to Figure 1, secondary structural elements are colored according to their conservation status in the overall superfamily as follows: yellow, elements are part of the “universal core” seen in all kinases in the superfamily; orange, elements are present in more than two, but not all, of the kinases in the superfamily; red, elements shared between only two families; purple, elements seen only in this family, but inserted within in the portion of the chain forming the universal core; blue, elements seen only in this family, and connected to the N- or C-terminal ends of the universal core. Secondary structural elements are labeled according to the standard conventions for the individual structure. As in Figure 1, the glycine-rich loop is rendered in green and the loop forming the linker region is rendered in red. For clarity, the conserved residues shown in Figure 1 are not rendered in these structures, though in most cases they are similar. Structures shown are as follows: (A) aminoglycoside phosphotransferase (APH(3′)-IIIa [24]); (B) CK (CKA-2 [23]); (C) ChaK [20]; (D) PI3K [21]; (E) AFK [22]; and (F) PIPKIIβ [19]. Molecular renderings in this figure were created with MOLSCRIPT [90].

With the acceleration in the rate of deposition to the Protein Data Bank (PDB) [18], a large complement of sequence-divergent TPK structures have become available, and make a more comprehensive structural study of this family possible. Additionally, several structures of distant TPK relatives have become available [19–24]. These atypical kinases (AKs) are phosphotransferases that clearly share homology with the TPK catalytic core, but do not conserve all of the usual kinase motifs, and modify the initial notions of the “essential” fold characteristics of protein kinase–like phosphotransferases. While they are termed “atypical” relative to the TPKs, the AKs often represent relatively large families of important proteins (an overview of the structures of the catalytic cores of the AKs is provided in Figure 2, and summary information is provided in Table 1).

**Table 1:**
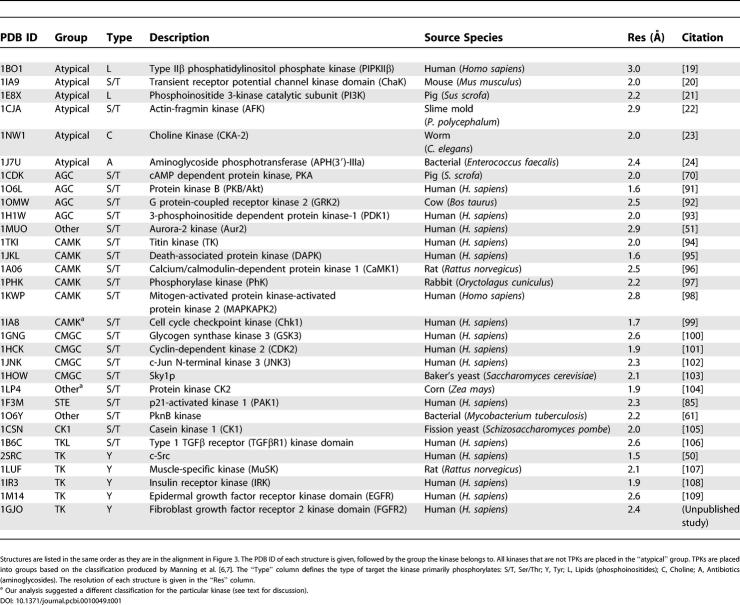
Kinase Structures Included in the Representative Set

**Figure 3:**
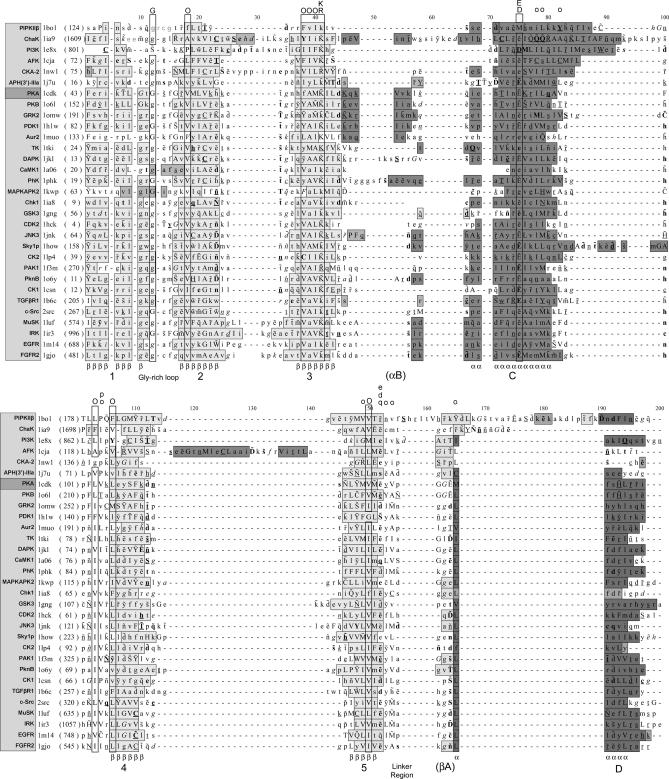
Enhanced Sequence Alignment Derived from the Structural Alignment of Kinase Representatives Abbreviated names of kinase representatives are provided with the gray box at the left-hand side of the figure (see Table 1 for more information on structures). The name is followed by the PDB ID [18] for the structure used in the alignment. The number in parenthesis following the PDB ID is the residue number of the first residue shown in the alignment. The sequences of the six AKs are clustered at the top of the alignment, followed by the sequence of PKA, which is highlighted. The alignment is annotated for key structural features using the JOY software [78]. Secondary structure is represented using the following conventions: light-gray box, β-strand; medium-gray box, 3–10 helix; dark-gray box, α-helix. Residue characteristics are represented using the following conventions: uppercase, solvent inaccessible; lowercase, solvent accessible; bold, hydrogen bond to main chain amide; underline, hydrogen bond to main chain carbonyl; tilde, hydrogen bond to other side-chain; italic, positive Φ; breve, *cis*-peptide. Residues that are highly conserved within the TPK family and some AKs are highlighted in boxes for the sequences where the conservation applies. The residue(s) seen at these positions are shown in uppercase above the boxes. The letter O stands for general hydrophobicity, but not a specific residue type. Residues that are more weakly conserved in the TPKs but are also conserved in many other AK families are noted with a lowercase letter above the appropriate alignment columns. Selected residues of interest that are conserved only within the TPKs are depicted using the same conventions above, but with gray lettering (depiction of residues conserved only in the TPKs is not exhaustive, i.e., only residues discussed in the text are highlighted above the alignment. Generally, this is done in structural regions unique to the TPKs). Secondary structures are labeled with the nomenclature used for PKA [12]. Sequence representing unresolved portions of the structure is not shown by JOY. In key portions of the alignment, this sequence is added back in and shown in light gray.

**Figure 3 (continued):**
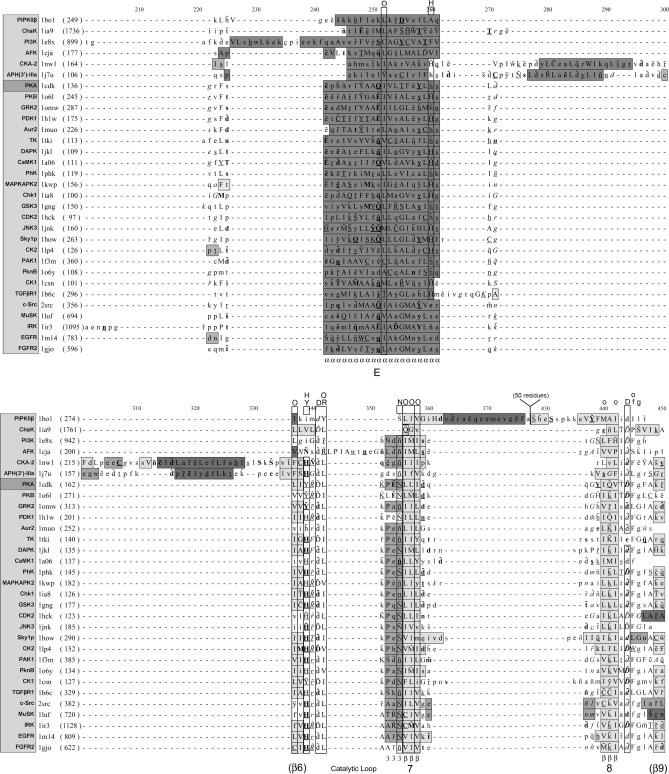
Enhanced Sequence Alignment Derived from the Structural Alignment of Kinase Representatives

**Figure 3 (continued):**
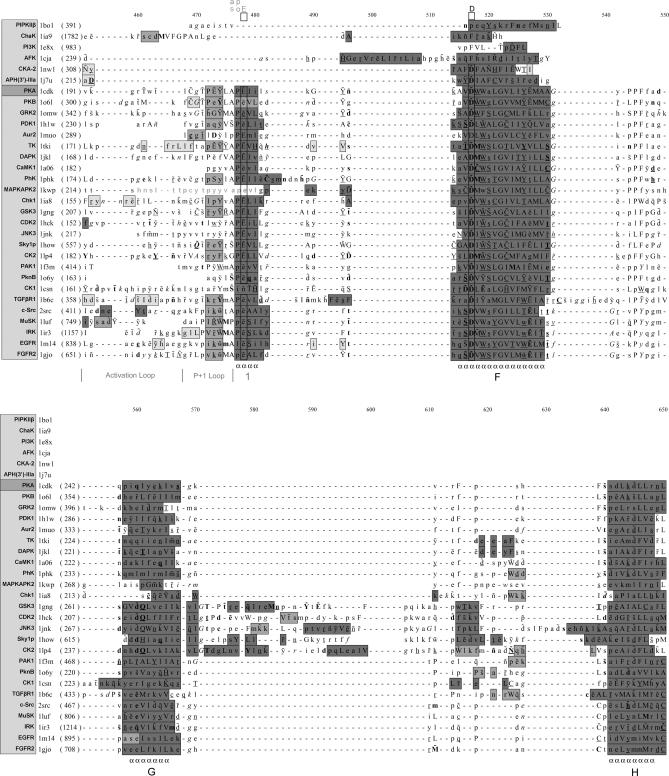
Enhanced Sequence Alignment Derived from the Structural Alignment of Kinase Representatives

**Figure 3 (continued):**
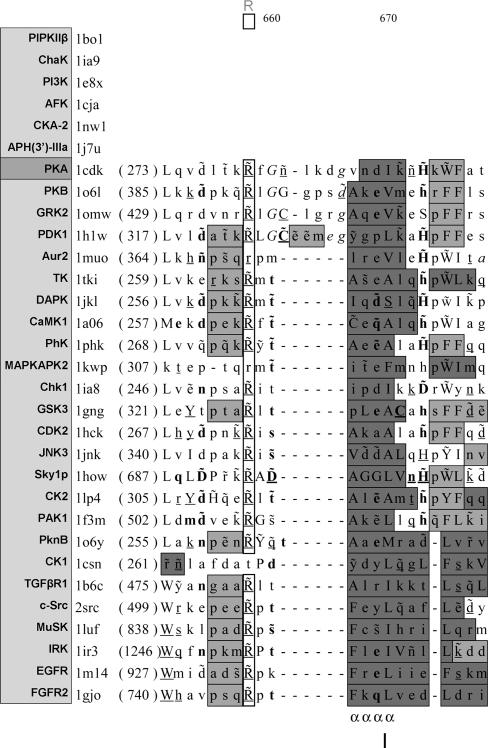
Enhanced Sequence Alignment Derived from the Structural Alignment of Kinase Representatives

The aminoglycoside phosphotransferase APH(3′)-IIIa is a kinase that phosphorylates several aminoglycoside antibiotics at the 3′ and/or 5′′ hydroxyl, inactivating them [25]. Though the structure of this enzyme has clear similarities to that of the TPKs, it also has distinct structural motifs, particularly in the C-terminal subdomain [4] (Figure 2).

Choline kinase (CK) participates in the pathway that eventually produces phosphatidylcholine, an important constituent of cell membranes that can be cleaved to produce a variety of second messengers [26]. The available structure is of choline kinase isoform A-2 (CKA-2) from *Caenorhabditis elegans* [23]. This structure has a very large and complex C-terminal domain, with features distinct from those of the TPKs (Figure 2).

Channel kinase (ChaK) is a protein kinase domain that is an integral part of a transient receptor potential channel. ChaK is a representative of the α-kinase family, a small but important kinase family that has no detectable sequence similarity to the TPKs [27]. The α-kinases are so named because they appear to phosphorylate residues within α-helices [28], as opposed to the loop-type regions targeted by the TPKs [29]. ChaK has a relatively similar N-terminal subdomain to that of the TPKs, but its C-terminal domain is extensively modified [20] (Figure 2).

Phosphoinositide 3-kinases (PI3Ks) phosphorylate various forms of phosphatidylinositol (PI) at the 3-hydroxyl position. The available PI3K structure [21] is that of PI3Kγ, a “class IB” PI3K that preferentially phosphorylates phosphatidylinositol 4,5-bisphosphate [PI(4,5)P_2_], creating phosphatidylinositol 3,4,5-trisphosphate [PI(3,4,5)P_3_] [30]. PI(3,4,5)P_3_ is an important second messenger that activates a variety of pathways in cells [31]. Relative to the TPKs, PI3K has a somewhat “flat-faced” architecture, with a more open active-site region (Figure 2). This structure allows it (in concert with accessory domains) to interact directly with the plasma membrane and phosphorylate PI in situ [21].

Actin-fragmin kinase (AFK) is a Thr protein kinase that has been isolated from the slime mold *Physarum polycephalum,* and at present has been detected in only this one organism. It phosphorylates actin when it is bound to the protein fragmin, helping to render control over actin polymerization [32]. Though this enzyme is clearly homologous to the TPKs, it has a modified N-terminal subdomain and an extensively modified C-terminal subdomain (Figure 2). The modifications in the C-terminal domain produce a flattened substrate binding region that allows for binding to the target actin molecule [22].

Type IIβ phosphatidylinositol phosphate kinase (PIPKIIβ) phosphorylates phosphatidylinositol 5-phosphate (PI5P) at the 4-hydroxyl position to generate PI(4,5)P_2_. PI(4,5)P_2_ is an important second messenger in cells [33], and can be further phosphorylated by PI3K as described above. The enzyme forms a homodimer that displays a highly flat-faced architecture with large patches of positively charged residues. This structure appears to allow PIPKIIβ to interact directly with the cell membrane, phosphorylating PI5P in situ [19]. PIPKIIβ is a structurally divergent enzyme that is not actually within the protein kinase–like superfamily as defined by SCOP. PIPKIIβ has almost no sequence similarity, and weak structural similarity, to the protein kinase–like superfamily. For this reason, it is in a different fold grouping in the SCOP hierarchy (d.143.1, as opposed to d.144.1). However, a careful study has linked this structure to the protein kinase–like superfamily through comparative structure analysis [34]. Cheek et al. have provided a comprehensive classification for all kinases, including the many superfamilies without any evolutionary relationship to the protein kinase–like superfamily (when the term “kinase” is used in this work, it refers specifically to members of the protein kinase–like superfamily) [35,36]. Unlike SCOP, they have placed the PIPK family within the same fold group as the kinase superfamily. Also, PIPKIIβ appears to share a similar catalytic mechanism to that of the kinases. Therefore, it is considered in this work, as an example of an evolutionarily ambiguous structural relationship.

We sought to use the structures of these AKs and the TPKs to determine a true “essential” kinase fold that is seen in all members of the kinase superfamily, as well as shared structural characteristics between the various families. We encoded these structural characteristics into a phylogenetic character matrix. We then combined this information with a structure-based sequence alignment in a unified Bayesian phylogenetic analysis [37,38]. Such an approach has been used previously for sequence data combined with morphological data, to determine relationships between species [39]. Also, discrete structural and sequence motif characters have been used previously to study fold-level relationships between protein structures [40]. However, to our knowledge, our study is the first in which the nuanced information available in a full-length sequence alignment is combined with structural characters in a unified analysis. Use of these two complementary sources of data allowed us to make rational phylogenetic predictions with high confidence, despite the very low sequence similarity inherent in superfamily-level comparisons. The results provide considerable insight into the development of the various kinases in the superfamily from a common ancestor. In addition, our approach offers a new and broadly applicable approach to the study of protein superfamily evolution.

## Results/Discussion

### Selection of a Representative Kinase Structure Set

The large number of kinase structures available necessitated the selection of a representative set of non-redundant structures for structural alignment. We used a rigorous framework based on both sequence and structural criteria to select the most representative structures within the superfamily. Our criteria were guided primarily by the structure classification provided by the SCOP database [1] (see Materials and Methods for details of our selection criteria). The resulting set of structures constituted 25 TPKs and the six AKs described in the introduction (Table 1).

### Structural Alignment and Analysis of the Superfamily

Creation of a highly accurate alignment using sequence information alone is difficult for the TPKs and impossible if the other superfamily members are included [41,42]. Therefore, in order to provide an overview of the structural and sequence features of the superfamily, we created an alignment of the structures based on their structural features. Although automated structure alignment methods are available [43], their accuracy is limited, and the ideal alignment of structures is often ambiguous [44,45]. Therefore, to ensure a highly accurate alignment the structures were aligned manually, using an automated multiple structure alignment as a starting point (see Materials and Methods).

Analysis of the aligned structures and sequences produced several key themes. First, the kinases all share a universal conserved core section, which roughly describes the region constituting the ATP binding pocket and locations of residues involved in the phosphotransfer reaction. Second, the conserved region, while mostly maintained in terms of its overall secondary structures, is often modified substantially in terms of the spatial placement of the structural elements. Third, the kinases generally have distinctive structural elements joined to both the N- and C-terminal ends of the universal core region. In addition, many also have substantial insertions that occur within conserved structural elements in the universal core region. In most cases, these structural insertions have absolutely no spatial similarity between families, though there are intriguing exceptions. Fourth, though the sequence similarity between families is very low, a small group of residues shows remarkable conservation across the entirety of the superfamily. Many of these residues have been previously recognized as highly important for proper activity in the TPKs [13,17]. Hence, it appears that the all of the kinases utilize a similar mechanism for phosphotransfer. The overall impression that emerges is one of a superfamily that has assiduously retained its basic function, but simultaneously has been heavily modified over the course of evolution to phosphorylate a variety of targets, interact with a range of partner proteins, and respond to different regulatory mechanisms.

### Phylogenetic Analysis of the Kinase Superfamily

Traditionally, molecular phylogenies are constructed as trees based on sequence similarity, coupled to an underlying theory of sequence evolution [46]. The extreme sequence divergence seen in the kinase superfamily (and in superfamilies in general) makes such determinations problematic. Therefore, in order to postulate an evolutionary history for the kinase superfamily, we constructed a phylogenetic tree using a Bayesian method [38,39] to integrate the sequence and structural data into a single analysis. This combined phylogenetic model provides higher reliability than a model produced using sequence or structural information alone.

Bayesian analysis was carried out using Markov Chain Monte Carlo as implemented in the program MrBayes [38,47]. The sequence alignment presented in Figure 3 was used as the input alignment. Because this sequence alignment was generated from a high-quality structural alignment, one difficulty normally posed when building trees for distantly related sequences—aligning them accurately—was eliminated. Hence, the only limitation on phylogenetic inference was the inherent sequence degradation at the superfamily level.

**Figure 4:**
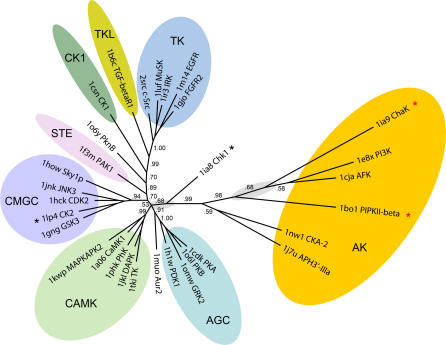
Proposed Phylogeny for the Kinase-Like Superfamily, Based on a Unified Bayesian Analysis of Both the Sequence Alignment in Figure 3 and the Structural Character Matrix in Table 2 Structures are labeled by their PDB IDs, followed by the abbreviated name of the structure. TPKs are to the left of the figure, and are labeled with their group membership. TPKs labeled with a black asterisk are classified differently in our tree compared with the classification produced by Manning et al*.* [7]. The AKs are highlighted with an orange oval. Major branches are labeled with their posterior probabilities. Gray ovals represent areas of doubt in the tree, based on the tree itself and other aspects of our analysis (see text). The left-hand oval represents uncertainty as to the closest TPK relative to the AKs; it is unclear where precisely the AKs should link to the TPKs (note that this uncertainty does not include the branching of most of the TPK groups in this region, as these are generally well supported). The right-hand oval represents uncertainty as to the proper placement of ChaK and PIPKIIβ. These kinases are difficult to place with high confidence because of their extreme divergence. They are labeled with red asterisks to denote the speculative nature of the current placement (see text).

Structural data were incorporated as a 20-column character matrix, containing the 20 distinctive structural characteristics described below (Table 2). Converting these characteristics into a character matrix allowed for much of the structural information from our comparative analysis to be quantitatively evaluated in MrBayes. These two datasets were simultaneously evaluated in MrBayes as “mixed” data, allowing for the creation of a single tree that provided maximum agreement with both (Figure 4; see Materials and Methods for detailed information).

**Table 2:**
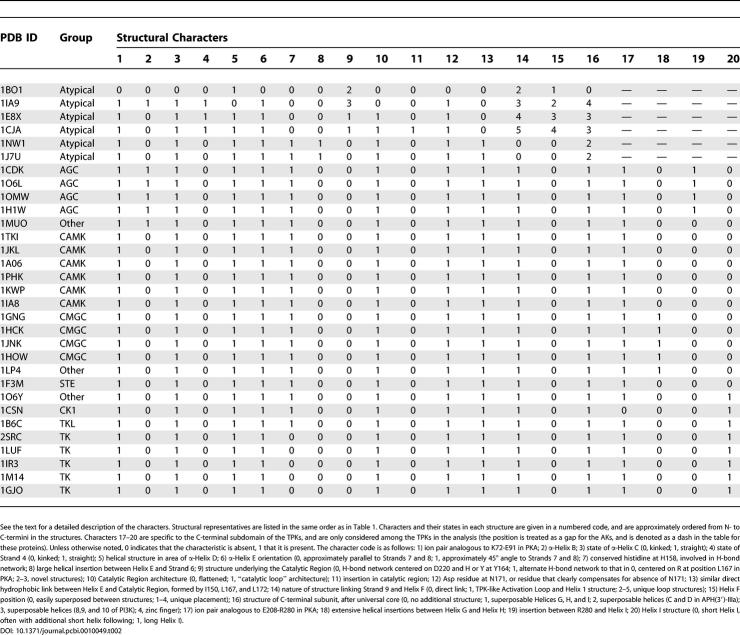
Distinctive Structural Characters Used in the Construction of a Phylogeny for the Kinases

### Selection of Structural Characters for Phylogenetic Analysis

Because protein structure is much more conserved than protein sequence over the course of evolution, it is possible to determine the likely relationships between proteins through comparative structure analysis. Structures that have similar features are likely to share a closer evolutionary relationship, especially if the features are uncommon in protein structures in general [34,40,48,49]. Based on our structural alignment, we undertook a careful comparative analysis of the structures in the superfamily to isolate distinctive structural characters seen in only one or more structures in the superfamily, but not all.

The majority of characters collected were in the universal core of the kinases, as this is the most conserved portion between the different families in the superfamily. This region represents a functional “cassette” responsible for the essential kinase functions of ATP binding and phosphotransfer. Almost all sequence and structure changes within this cassette during evolution would be expected to be deleterious to proper kinase function. Hence, in the most parsimonious scenario, any successful changes in the region would likely occur only once, and then be reused by progeny kinases. Therefore, similarities (and differences) seen within the universal core are expected to be more significant than similarities in other parts of the structures.

In addition, characters were collected for structures outside of the universal core shared by only a subset of the superfamily. Since these sorts of structures are further from the functional core, they can be expected to change more quickly than those within the core. Therefore, to be included, these sorts of structures had to be substantial and distinctive, as opposed to the more subtle structural differences accepted in the core. Finally, a subset of characters specific only to the TPKs was collected. Because there is more than one structure available for this family, this information was used to help improve the phylogenetic analysis within the highly diverse TPK family.

Since sequence motif information is inherently present in the sequence alignment (and this was included in the analysis), the presence/absence of particular sequence motifs was generally not included in the character matrix. However, specific modifications involving sequence that had special structural or functional implications were included, since in many cases the critical importance of these changes is not sufficiently expressed within the sequence data.

We provide a brief summary of each of the characters included in the analysis, and their importance to the structure and function of the enzymes. For the sake of economy, when secondary structural elements that form the universal core are named generically, we use the conventions used for protein kinase A (PKA) [12] (and many other TPKs) and use uppercase to denote this standardized nomenclature (e.g., “Helix C”). When elements from specific structures are discussed, the corresponding element names for these structures (where different from those for PKA) are provided in lowercase. Conversion of this scheme to that used for the other kinase families is available in the labeling of elements in Figures 1 and 2. Similarly, the residue numbers for generic residue positions are based on the residues and numbers for PKA. In cases where a residue number is provided that is specific to a structure, it is followed by the residue number for the comparable residue in PKA in parentheses (e.g., “Q1767(L172)”). Comparable residues for any other structure in the set may then be retrieved from the alignment provided in Figure 3. The characters are presented in approximate N-terminal to C-terminal order.

#### 1: Ion pair analogous to K72-E91 in PKA.

In all of the kinases, a very highly conserved lysine (K72) or arginine residue is present in Strand 3, facing the binding pocket. In most of the structures with bound ATP, K72 interacts with the α and β phosphates of the ATP molecule, helping to stabilize them in the proper conformation for phosphotransfer [15]. The position of K72 is stabilized by the formation of an ion pair with a glutamic acid residue (E91) in Helix C. By linking Helix C to Strand 3, the Lys-Glu ion pair also helps to stabilize the overall fold of the N-terminal subdomain. Some of the AKs have conservative substitutions at either of these positions (Figure 3). In others, such as PI3K [21] and ChaK [20], the negatively charged residue at E91 may play a diminished role, or form an ion pair with K72 only when the kinase is in an active conformation. Such conformational shifts are seen in the TPKs, wherein the K72-E91 ion pair is broken by movement of Helix C when the kinase is in an inactive state [15,50]. The one distinctive exception is seen in PIPKIIβ, which retains K72 but lacks a clear replacement for E91. D156(H87) in PIPKIIβ may fulfill the role of E91 in PKA [19], but unlike the other kinases, a negative charge has been completely removed from position E91 in PIPKIIβ.

#### 2: α-Helix B.

Between Strand 3 and Helix C, most of the kinases have a short loop structure. However, the AGC group of TPKs (Table 1) and the aurora-2 kinase [51] share the distinctive α-Helix B at this location (Figure 3). This helix is not seen in any of the other TPKs. Remarkably, however, it is seen in ChaK, where it is the same length, though it is shifted spatially from what is seen in the AGC kinase PKA (Figures 1–3). Hence, the conservation of Helix B in ChaK is surprising, particularly given its distinctive structure.

#### 3: Kink in α-Helix C.

In PIPKIIβ, helix 4 (Helix C) contains a distinctive kink not seen in any of the other kinases (Figure 2). This kink requires some reorganization of the ATP binding pocket and allows for interaction of the N-terminal subunit with the highly modified shape of the C-terminal subunit (see characters below). The kink also appears to play a role in the lack of a K72-E91 ion pair (character 1) in this structure, because it places the region of the helix where the required Glu residue would reside far from K150(K72).

#### 4: Kink in Strand 4.

Most kinases in the superfamily have a distinctive kink near the beginning of Strand 4. This kink modifies the placement and architecture of much of the hydrophobic pocket formed by Strand 4, Helix C, and Helix E. ChaK, PI3K, and AFK are the exceptions, and contain a straightened (and/or shortened) Strand 4 (strand 9 in ChaK; strand 6 in PI3K), which changes the architecture in this region of the core. This change results in the requirement for a gap within the Strand 4 region when aligning these structures with others in the superfamily (Figures 2 and 3).

#### 5: Helical structure in the area of α-Helix D.

Helix D appears just after the linker region in the TPKs (Figure 1). In most of the AKs, helical structures are present in this region, though they are not always superposable, and some are 3–10 helices rather than α-helices. However, ChaK is distinctive in that it completely lacks this element (Figure 2).

#### 6: Orientation of α-Helix E.

Helix E stabilizes the ATP binding pocket through its interactions with Strands 7 and 8. In most of the kinases, it is oriented at approximately 45° to these elements, but in PIPKIIβ, helix 6(Helix E) is approximately parallel to them, a major reorganization of the supporting structure of the catalytic core (Figure 2).

#### 7: Key conserved histidine at H158.

**Figure 5:**
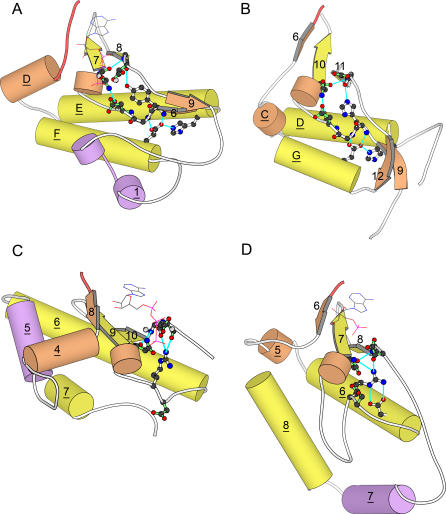
Shared Hydrogen-Bonding Networks between Distantly Related Structures in the Kinase-Like Superfamily Colors and nomenclature for secondary structural elements are identical to those provided in Figure 2. Structures shown are the C-terminal subdomains of four structures: (A) PKA [70]; (B) CKA-2 [23]; (C) PI3K [21]; and (D) AFK [22]. For clarity, some portions of structures are omitted. Residues involved in the shared hydrogen-bond networks are shown in a ball-and-stick rendering. For clarity, side-chains are omitted for residues that only participate in the network via backbone interactions. Residues involved directly in catalysis or metal binding are shown with light-green stick regions in the ball-and-stick rendering. Metal atoms, when present, are shown as gray spheres. ATP (or ATP analog), when present, is shown in a line rendering. Hydrogen bonds are shown in cyan. The orientation of the structures is similar but not identical (structures were rotated somewhat to make H-bond contacts more visible). Molecular renderings in this figure were created with MOLSCRIPT [90].

Helix E (helix D in CKA-2; helix 4 in APH(3′)-IIIa) also contains a conserved histidine residue, H158, which is shared only between the TPKs and the APH and CK families. Remarkably however, H158 is not conserved in the tyrosine kinase group within the TPKs. H158 forms a hydrogen bond with D220 and in so doing, participates in a hydrogen-bond network that links together Helices E, F, and the crossing loops in the catalytic region of these kinases (see below and Figure 5). Hence, in the conservation of this interaction, the APH and CK families display a closer relationship to the Ser/Thr TPKs than do the tyrosine kinases (it should be noted that H158, while conserved in APHs, is less conserved than it is in the Ser/Thr TPKs and CKs, and may be of somewhat reduced importance in this family).

#### 8: Large helical insertion between Helix E and Strand 6.

Two of the kinases, CKA-2 and APH(3′)-IIIa, contain a distinctive insertion immediately after Helix E (helix D in CKA-2; helix 4 in APH(3′)-IIIa). The shared insertion consists of two interacting helices, linked by a short loop containing a small helix (Figure 2). In both kinases, these insertions effectively replace the Activation/P+1 Loop of the TPKs (see character 14). Though they do not align perfectly (Figure 3), the striking similarity of these elements, and their absence in all other kinases, suggests that they are a product of relatively close common ancestry between CKs and APHs.

#### 9: Structure underlying the catalytic region.

The Catalytic Region of many of the kinase families is supported by complex hydrogen-bond networks that stabilize the architecture of the active site. There are distinctive similarities in these networks that suggest relatively close evolutionary relationships between some families. The TPKs, CKA-2, and APH(3′)-IIIa all share an H-bond network centered around a highly conserved His or Tyr residue at position Y164, which usually forms a hydrogen bond with the backbone carbonyl of position T183, just after the end of Strand 8 (strand 11 in CKA-2). This interaction is significant, because D184 is highly conserved, and interacts with a magnesium atom in the active site that is important for ATP interaction and the phosphotransfer reaction [13]. In addition, this region is the area in which a “crossing loops” structure is formed, where the catalytic loop and the loop between Strands 8 and 9 cross. This type of motif is unusual in protein structures, and is one of the hallmarks of the kinase superfamily [34]. The Y164-T183 hydrogen bond is also a part of a larger conserved H-bond network shared by the APH, CK, and TPK families. This network includes H158 in Helix E (character 7) and D220 in Helix F (helix G in CKA-2; helix 5 in APH(3′)-IIIa), and essentially ties together the catalytic region in these kinases (Figure 5).

In AFK and PI3K, the H-bond to the backbone of position T183 is instead made by an arginine residue at position L167 (Figures 3 and 5). This Arg residue effectively replaces, from a location three positions down the chain, the function of Y164. Thus, these two structures share a distinctive interaction at the center of their catalytic regions that replaces a conserved interaction seen in many of the other kinases. Further, these two kinases both lack the extended H-bond network seen in the three families above.

ChaK and PIPKIIβ do not have any of the H-bonding patterns seen in the other two groups. They each use unique underlying structures to stabilize their catalytic regions.

#### 10: Architecture of the catalytic region.

Between a highly conserved Asp (important for catalysis) at position D166 and Strand 7 the backbone in most of the kinases adopts a structure commonly called the “catalytic loop.” In most structures containing the element, this “loop” actually consists partly of a short 3–10 helix. Two structures, PIPKIIβ and ChaK, lack the catalytic loop completely, and instead have an approximately linear connection between D166 and Strand 7 (strand 10 in PIPKIIβ; strand 13 in ChaK; Figures 2 and 3).

#### 11: Insertion in the catalytic region.

Following the Arg residue at position L167, AFK contains an insert that loops away from the catalytic region and interacts with the C-terminal subdomain. This element is unique to AFK (Figure 2).

#### 12: Asp residue at 171, or apparent compensation for its absence.

In those structures containing the 3–10 helix (or a loop in a similar conformation), the last position of the helix contains a highly conserved asparagine residue, N171. This important residue is responsible for interaction with a magnesium ion, which in turn interacts with the phosphate groups of ATP [13]. It also participates in the H-bond network discussed above (see “Structure underlying the catalytic region”), further increasing its importance (Figures 3 and 5).

In the two kinases lacking the helical element, there is an interesting divergence in compensation for the lack of N171. In ChaK, the next position down the chain, Q1767(L172) is the highly similar residue glutamine. Remarkably, the longer side-chain of this glutamine is angled such that the amide group is in a similar location in space to the amide group of N171 in the other structures. Conversely, in PIPKIIβ there is no obvious compensation for the loss of N171, and since ATP is not present in this structure it is unclear how PIPKIIβ interacts with ATP without N171. Hence, ChaK is more similar to the rest of the kinases in this area of the structures, and this is reflected in our matrix (Table 2).

#### 13: Similar direct hydrophobic link between catalytic region and Helix E.

In the structures of the TPKs, APH(3′)-IIIa, and CKA-2, conserved hydrophobic residues (L167 and L172) flank the 3–10 helix and face into the hydrophobic core. They interact directly with each other, as well as a conserved hydrophobic residue at I150 in Helix E (helix D in CKA-2; helix 4 in APH(3′)-IIIa). Though many other kinase families have conserved hydrophobic residues at these positions (Figure 3) and have a clear hydrophobic pocket, this distinctive link is specific to the TPK, APH, and CK families. These interactions are important because they form a direct link between the Catalytic Loop and Helix E, stabilizing the conformation of the Catalytic Loop.

#### 14: Nature of structure linking Strand 9 and Helix F.

The region immediately following Strand 9 is termed the “Activation Loop” in the TPKs, because many TPKs are regulated by phosphorylation of residues in this loop [15,52–54]. All of the TPKs in our set have a substantial activation loop (Figure 3). The loop immediately following the Activation Loop is often termed the “P+1 loop” in the TPKs, because it interacts with residues in the substrate protein chain one position (and beyond) from the actual residue targeted for phosphorylation [29]. The P+1 loop is followed by the distinctive APE (or similar) motif in most TPKs. Beginning at P207 in the motif there is a conserved helix, which we term Helix 1 to avoid conflict with the standard TPK naming scheme. The last residue in the APE motif, E208, is highly conserved within the TPKs. It forms an ion pair with an arginine residue, R280, further down the chain. R280 is located in a loop between Helices H and I. Hence, the effect of the ion pair is to hold the C-terminal subdomain together. This ion pair is retained in all TPKs except the CK1 group (see character 17). However, in terms of overall architecture, all the TPKs have a similar structure in the Helix 1 region (and the rest of the C-terminal subunit).

None of the AKs share a similar structure to TPKs in the Activation Loop region (Figure 2). Most structures have a markedly shortened loop relative to that seen for the activation/P+1 loops in the TPKs, and the structures are distinct in most families (accurate analysis of the Activation Loop regions of many of the AKs is difficult because they are not resolved in the experimental structures). The exceptions are CKA-2 and APH(3′)-IIIa, which share a distinctive short and highly twisted β-sheet in the Activation Loop region formed by Strands 6 and 9 (strands 9 and 12 in CKA-2; Figure 2). This structure allows for an extremely short “Activation Loop,” the shortest within the superfamily.

#### 15: Positioning of Helix F.

Helix F, which follows the various loop structures, constitutes the last region of structural similarity shared by all of the kinases, though the similarity in this region drops off rapidly. It could be argued that in some cases, this helix superposes so poorly between superfamily structures that it should not be considered part of the “universal core.” However, it is present with an approximately similar orientation in all structures, and in most cases seems to have a similar role: stabilization of the backbone of the Catalytic Loop. However, the manner in which this stabilization is achieved is highly variable.

An exception to this variability is seen between the TPKs, APH(3′)-IIIa, and CKA-2. In these three families, Helix F (helix G in CKA-2; helix 5 in APH(3′)-IIIa) is maintained in a highly similar orientation and is readily superposable (Figures 1 and 2). More significantly, the families share an aspartate residue, D220, that is highly conserved in the three families. This residue forms hydrogen bonds with the backbone amides of Y164 and R165 and (with the exception of the tyrosine kinases; see character 7 above) the side-chain of H158. Hence, a network of residues and contacts that is responsible for the specific geometry of the most conserved regions of the kinase fold has been carefully conserved in these three kinase families.

Though Helix F can be superposed relatively well between the TPK, APH, and CK families, it is much more variable in the four remaining families, and is only weakly superposable. The large helical insertion into the Activation Loop of AFK pushes helix 8 (Helix F) into an angled position, such that it tilts away from the catalytic loop. The space opened by this translocation is filled by the insertion seen in the middle of the catalytic loop in this structure (character 11 and Figure 2). In PI3K, helix 7 (Helix F) is shortened such that a loop region interacts with much of the catalytic loop, partly replacing the role of Helix F in other structures (Figure 2). In ChaK, helix E (Helix F) is shortened and tilted away from the catalytic loop to the point that it appears to play no direct role in stabilizing this element. PIPKIIβ has a structure that is more similar to what is seen in Helix F in the TPKs, except that the orientation of helix 8 (Helix F) relative to strands 10 and 12 (Strands 7 and 8) is nearly parallel, rather than an approximate 45° angle as seen in the TPKs (Figure 2).

#### 16: Structural similarities in C-terminal subunit, following the universal core.

Though Helix F represents the end of the universal core shared by all kinases in the superfamily, many of the kinases have additional structure beyond this point, and there are shared substructures between some families that argue for a closer evolutionary relationship. All of the TPKs share superposable Helices G, H, and I (Figures 1 and 3). However, none of the other kinase families contain these structures.

APH(3′)-IIIa and CKA-2 share two superposable helices in their C-terminal subunits along with a very similar overall topology. CKA-2 follows helix G (Helix F) with a small β-sheet and a small helix, which APH(3′)-IIIa lacks. However, the helix that follows is superposable between the structures. After this helix, CKA-2 has an additional two helices, while APH(3′)-IIIa has an irregular loop structure. However, the overall path of the chain is identical between the two structures, and they share another superposable helix in the likely substrate binding region. The chain of APH(3′)-IIIa terminates at the end of this helix, while CKA-2 adds an additional two helices (Figure 2).

AFK and PI3K have differing structures in the area of Helix F (helix 8 in AFK; helix 7 in PI3K). However, immediately following this region the two structures share a set of similar helical elements. The first of these helices interacts with Helix E (helix 6 in both AFK and PI3K), and superposes well between the two structures. The second and third of these helices superpose only weakly. However, they are in approximately similar orientations, and together with the first helix form a motif that is distinct within the superfamily. After the third helix, PI3K has two additional helices, which are not seen in AFK (Figure 2).

The C-terminal subdomain structure of ChaK is completely novel, and not shared by any other kinase in the superfamily. Remarkably, a zinc finger [55] forms the center of the subdomain and links all the major elements together [20]. The zinc coordination links helices D and E (Helices E and F) and the final terminal helix, which each provide one of the coordinating histidine or cysteine residues. The final coordinating cysteine is provided by the loop linking helix E and the final helix.

The C-terminal subdomain of PIPKIIβ contains essentially no additional structure beyond helix 8 (Helix F).

#### 17: Ion pair analogous to E208-R280 in PKA (TPKs only).

In CK1, the APE sequence in Helix 1 (described above) is replaced with the motif SIN (which is conserved within the CK1 group). This motif essentially fills the roles of APE in the first two positions, but at position N188(E208), an asparagine residue replaces the glutamate seen in other TPKs, and hence no ion pair is formed. CK1 also does not contain a positively charged residue that correlates to R208 in the other TPKs (Figure 3). However, it substitutes a new ion pair that the other TPKs lack. Residue E202(W222) from Helix F forms an ion pair with residue R261(L273) from Helix H. Thus, the linkage between different regions of the C-terminal subdomain is essentially retained, albeit with a pair of residues that are novel with respect to the rest of the TPKs. The substitution of APE with SIN (and a different ion pairing) may have implications for the evolution of CK1 relative to the other TPKs, given the strict conservation of the E208-R280 ion pair in these structures. However, the overall structure of the C-terminal subdomain of CK1 is still very similar to that for the other TPKs.

#### 18: Extensive helical insertions between Helix G and Helix H (TPKs only).

The CMGC group of TPKs contains distinctive helical insertions between Helix G and Helix H. These insertions are variable in position and helix length, but they are much more extensive than the small insertions occasionally seen in other families. Interestingly, CK2 also contains these insertions (Figure 3).

#### 19: Insertion between R280 and Helix I (TPKs only).

The AGC kinases share a distinctive insertion between R280 and Helix I (Figure 3).

#### 20: Helix I structure (TPKs only).

Helix I often actually consists of two shorter helices joined by a linker. In most cases, the first helix is an α-helix, and the second is a 3–10 helix (Figure 3). This split helix structure is dominant for Ser/Thr kinases, while Tyr kinases have a single long Helix I. Interestingly, three Ser/Thr kinases share the Tyr kinase–like architecture for Helix I. One of these is TGFβR1 from the tyrosine kinase–like (TKL) group, so the structural similarity is unsurprising. However the other two kinases, CK1 and the bacterial kinase PknB, do not have an obvious reason to display this similarity to the Tyr kinases.

### Comparing the Phylogenetic Analysis with Other Data

We interrogated our phylogenetic model against the backdrop of species distribution of the families. We utilized the pre-computed results available in PFAM [56] to survey the presence or absence of the kinase families corresponding to structures in our set in the three superkingdoms of life (Table 3). These species representation data also fit well with other lines of inquiry (see below). We also created superpositions of selected structures based on our alignment to provide root mean square deviation (RMSD) values as a general estimate of structural similarity (Table 4). These were helpful in augmenting our own qualitative knowledge of structural similarities seen between the families, and their likely significance.

**Table 3:**
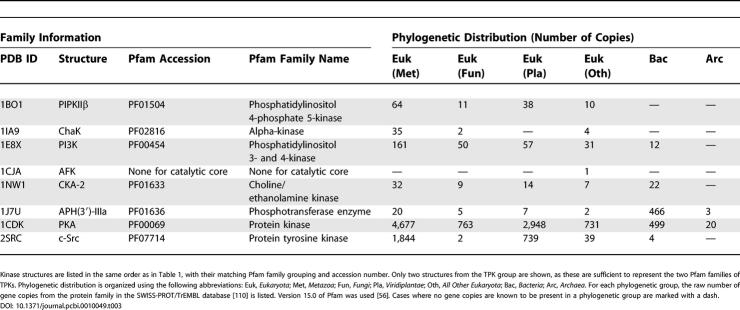
Phylogenetic Distribution of Kinase Families within the Superfamily, According to the Pfam Resource

**Table 4:**
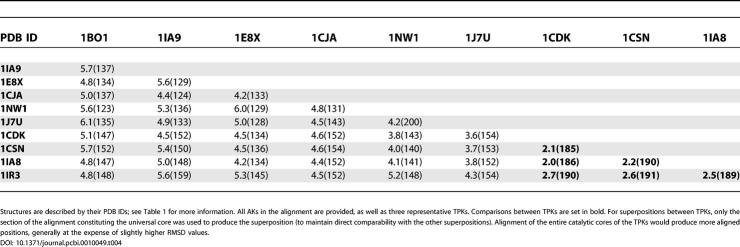
RMSD and Number of Aligned Residues from Representative Kinase Structure Alignments, When Superposed Based on the Alignment Presented in Figure 3

**Figure 6:**
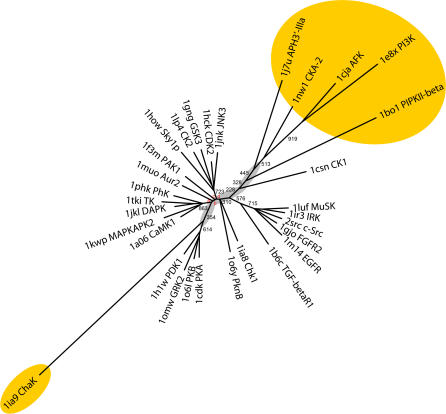
Conventional Distance-Based Phylogenetic Tree of the Kinase-Like Superfamily, Based Only on the Sequence Alignment from Figure 3 This tree did not explicitly incorporate structural information, and is provided for purposes of comparison with the Bayesian tree presented in Figure 4. Structures are labeled by their PDB IDs, followed by the abbreviated name of the structure. The AKs are highlighted by orange ovals. Bootstrap values are provided for major branches. Some branches are too short for values to fit; these are marked with red letters that correspond to the following values: a, 199; b, 170; c, 101; d, 141. Branches highlighted in gray were not supported by bootstrap values above 500, and should be considered speculative (if based only on this tree data) [57,58]. Many of the core relationships within the superfamily cannot be resolved with confidence using the conventional sequence-based approach.

Finally, we compared our tree with a tree made using only sequence information and a more traditional distance-based method of phylogenetic inference, to provide a comparative benchmark (Figure 6; see Materials and Methods for details of the tree construction). Although this tree did not utilize structural information, it still could take advantage of the highly accurate sequence alignment. However, this tree demonstrates the difficulty inherent in using sequence information alone to discern superfamily-level relationships. While the tree is able to successfully cluster groups of similar proteins out at the edges with acceptable confidence, the center of the tree suffers from low bootstrap values, and thus is somewhat speculative in these areas (we report branches with bootstrap values of < 50% of replicates as speculative based on the results of benchmarking studies [57,58]). Interestingly, comparison with the tree produced with MrBayes reveals a large degree of overlap. Areas of agreement between the two trees provide additional supporting evidence for the validity of the results.

However, we believe that the MrBayes tree is much more reliable than the conventional tree, given the explicit addition of structural information. Review of Bayesian trees generated using only the sequence information or structural information (Figures S1 and S2) demonstrated that neither dataset alone was capable of producing a resolved tree. When compared with the tree generated by sequence alone, the tree incorporating structural information (presented in Figure 4) provided several concrete benefits. First, the combined tree resolved polytomies (“star trees” at particular nodes) seen in the sequence-only tree. Second, the combined tree provided higher branch confidence values for many branches (in Bayesian trees, branch confidence values are estimated as posterior probabilities, which are generally interpreted as the probability that a branch is correct, provided that the evolutionary model and priors are correct [37,59]). Third, where branch changes occurred in the combined tree, the net effect was generally to produce a tree with better agreement with the structural observations (i.e., the use of structural characters in the analysis produced the desired effect). We discuss the various data within the context of the implications for structural evolution of the kinases.

### How Did the Various Kinases Evolve?

#### The TPKs appear to be ancient but display remarkable conservation of sequence and structural features.

Against the backdrop of the AKs, the TPKs can be seen to be a remarkably well-conserved family of enzymes, given their high level of duplication and broad distribution within the three superkingdoms of life (Table 3) [6,8–11,60]. It would appear that the TPK core structure, once arrived at, has required little modification in order to switch to many different protein substrates. The TPKs share not only a highly similar core cassette but also a large amount of distinctive substrate binding and stabilizing structure in their C-terminal regions. In addition, they contain numerous sequence motifs that are extremely well conserved, even though some (such as APE) appear to play a primarily structural role that would seem to be replaceable with other sequences and structures. Consistent with these observations, the TPKs form a relatively tight cluster in our phylogenetic tree, with clear subsections representing the Ser/Thr kinases and Tyr kinases, and the TKL group as an intermediate between the two (Figure 4).

Interestingly, our tree also places the one bacterial TPK structure available, PknB [61], in its own distinct group near the center of the tree, in the middle of the radiation of the TPKs (Figure 4). This location is consistent with a scenario in which an ancestor of the TPKs arose before the radiation of the three superkingdoms of life, with the other TPKs in our tree developing separately in eukaryotes. Leonard et al. have conducted an in-depth sequence-based study that placed the PknB kinase within the “Pkn2” group of kinases in bacteria, and noted that of the prokaryotic kinases, the Pkn2 group is the most closely related to the TPKs seen in eukaryotes [8]. Pkn2 kinases are not seen in archaea, and Leonard et al. suggested that this indicates that the Pkn2 group was horizontally transferred into bacteria from eukaryotes shortly after the divergence of the three superkingdoms of life. Thus, some of the eukaryotic-like TPKs seen in bacteria could be the result of an early horizontal transfer event. Our tree would also be consistent with this scenario. It should be noted that any scenario for the development of TPKs in bacteria must place them into the bacterial lineage very early in evolution, given their very broad distribution in this superkingdom [8,9,11,60], and results of codon bias and G/C content studies [62].

Manning et al*.* have produced a tree for the all TPKs in the human genome, using sequence information only [7]. As our tree had the benefit of a potentially more accurate sequence alignment, as well as the inclusion of structural features, we sought to compare our results with theirs. The two trees display a high level of agreement, though some differences are evident. Interestingly, where our tree differs substantially, we are often able to offer structural arguments suggesting that our tree is more likely to be correct.

In terms of the overall tree architecture of the various TPK groups, our tree is nearly identical to that by Manning et al*.*, with the exception that their tree places the STE group kinases closer to the TKL and TK groups than the CK1 group. Our tree places the CK1 group closer to TKL/TK than STE, with a very high posterior probability (Figure 4). As noted above, the TK, TKL, and CK1 groups share a similar Helix I structure that is changed in all other eukaryotic TPKs in our set (Table 2, character 20, and Figure 3).

We also classify two specific kinases differently than Manning et al*.* The first, CK2, is classified by Manning et al*.* as “other” and placed near the root of the CMGC group on their tree. Our tree instead places CK2 well within the CMGC group, with a high posterior probability on the major branch separating the group from the rest of the TPKs (Figure 4). As described above, CK2 also contains the distinctive helical insertions between Helices G and H, insertions otherwise only seen within members of the CMGC group (Table 2, character 18). Finally, our conventional tree also places CK2 well within the CMGC group, with a reasonably strong bootstrap value for the major branch (Figure 6). We submit that CK2 should be considered fully a member of the CMGC group. The other kinase for which our classification differs is cell cycle checkpoint kinase (Chk1). Manning et al*.* classify this kinase as a member of the CAMK group, placing it near the root of the group. Our tree classifies this kinase as “other,” and the separated CAMK group has a very high posterior probability on its main branch, indicating that the rest of the CAMK group is very sequence distinct from Chk1 (Figure 4). Our conventional tree also separates Chk1 from the CAMK group, with a strong bootstrap value separating the CAMK group from Chk1 and the rest of the TPK family (Figure 6). However, in this case there is no direct structural argument for the placement of Chk1 in or out of the CAMK group. Therefore, we remove Chk1 from the CAMK group for purposes of our analysis, but do not necessarily argue for its reclassification.

#### The TPK that forms the closest link with the AKs is difficult to determine.

The AKs form a distinct phyletic group (see below), but the TPK that constitutes the closest link to the AKs is difficult to verify with a high degree of certainty. Our tree places Chk1 in this position, with a moderate posterior probability (Figure 4). Chk1 does seem to potentially be a good candidate, as it is widely distributed in eukaryotes, and is a key player in the critical (and presumably ancient) cellular response to DNA damage, as well as cell cycle control [63].

However, there is no compelling structural evidence linking Chk1 to the AKs. Only two of our structural characters show partial representation in both the TPKs and AKs, thus providing structural information as to possible TPK/AK links (characters 2 and 7; see above and Table 2). These two characters do not directly link Chk1 to the AKs. Chk1 also does not show any tendency toward lower RMSD values when aligned to the AKs, relative to other TPKs (Table 4). Hence, the linking of Chk1 to the AKs is done primarily through sequence, which can be unreliable at this level of divergence.

Given this level of doubt in the analysis, it is not surprising that our conventional tree instead presents CK1 as being the closest link (Figure 6). Bootstrap support is very weak for the link, but as with Chk1, CK1 does have some characteristics that make it attractive as the link to the AKs. CK1 is the only kinase to replace the APE motif with a SIN motif, and in the process lose the distinctive E208-R280 ion pair seen in other TPKs (see above). As the AKs obviously lack this ion pair as well, CK1 could be seen as a more “primitive” kinase. Given the very broad distribution of the CK1 group in eukaryotes [6], the ion pair switch appears likely to have occurred shortly after the separation of eukaryotes into a distinct superkingdom. CK1 also has a variety of other sequence peculiarities that cause it to be placed in a unique location on our phylogenetic trees, intermediate between the Ser/Thr kinases and Tyr kinases (Figures 4 and 6). Hence, CK1 likely represents an ancient group of TPKs.

However, we are not aware of any confirmed case of a CK1-like kinase in prokaryotes, indicating that CK1-like kinases are limited to eukaryotes. BLAST searches by us against all bacterial genomes revealed that the 50 highest scoring hits (BLAST E-values from 2 × 10^−14^ to 1 × 10^−8^) maintained the usual APE motif seen in the rest of the TPKs (or similar motifs seen in the TPKs, such as SPE). Further, the changes seen in CK1 are relatively minor compared with differences between the TPKs and the AKs, and our structural analysis did not indicate any direct evidence that the CK1 group should be considered closely linked to the AKs. Though CK1 is missing the APE motif, it still has a P+1 loop and Helix 1 structure that are very similar to the other TPKs (Figure 3). CK1 also does not align to the AKs with lower RMSD or more aligned positions, relative to the other TPKs (Table 4).

The examples of Chk1 and CK1 illustrate the difficulty in determining the specific TPK that constitutes the closest link to the AKs. Though Chk1 appears to be the strongest candidate at this time for the closest link to the AKs, we believe that such links will remain speculative in the absence of new kinase structures that might provide additional insights.

### The AKs Form a Distinct Group

There is strong evidence that the AKs form a separate phyletic group, and that this group has an ancient origin, probably evolving as early as the TPKs. This is in contrast to an alternate scenario where the TPKs developed first and then the AKs arose via intermittent divergence from various TPKs. An ancient origin for the AKs is supported by our tree, which separates the AKs from the TPKs completely, with a very high posterior probability on the separating branch (Figure 4). Three of the families, the PI3Ks, CKs, and APHs, are broadly distributed in eukaryotes and seen in many bacteria, similar to the pattern seen in the TPKs (two AK families, the PIPKs and α-kinases, are not so broadly distributed and have a more puzzling origin; see next section). This is the opposite pattern from what would be expected if these AKs had diverged intermittently, in which case they would appear in only a subset of organisms. These three AK families traverse the entirety of the AK portion of the tree, helping to establish its ancient origin. Further, as mentioned in the previous section, only two of our structural characters indicated that specific AK families might have closer relationships with specific TPK groups. In other words, most of the AKs do not appear to simply represent different modifications of extant TPK structures.

Within the AKs, the CKs and APHs can be most closely linked with the TPKs. These three families share distinctive structure and sequence motifs within their core cassettes that stabilize the geometry of the catalytic residues and the crossing loops (see structure analysis above, and Table 2). Also, it has been shown that APH(3′)-IIIa has some protein kinase activity [64], providing a functional link between the APHs and TPKs.

As stated previously, CKA-2 and APH(3′)-IIIa also share a remarkable amount of additional structure within their C-terminal subdomains (Figure 2). This structure is seen in two different sections of the protein chain, extensive in length, superposable, and not seen in any other member of the superfamily. These observations argue compellingly that the CK and APH families are relatively closely related, and the most closely related within the superfamily. Accordingly, our phylogenetic tree places APH(3′)-IIIa and CKA-2 close together, though with considerable evolutionary distance after their split (Figure 4). It would appear that choline and APHs shared a similar common ancestor. This common ancestor, in turn, shared a relatively close common ancestor with the TPKs. Whether the common ancestor looked more like a TPK or the APHs/CKs is unknown.

The TPK/APH/CK cluster can be linked to PI3K and AFK partly by establishing a major evolutionary split in the superfamily based on the structure of the core cassette. Most of the families within the superfamily have a short 3–10 helix (or a loop in nearly this conformation) in the middle of their catalytic loop regions. In all of these structures, the third position of this 3–10 helix contains a highly conserved asparagine residue, N171, which is responsible for binding a metal ion. In addition, this 3–10 helix is nearly immediately preceded by the most highly conserved reside in the superfamily, D166 (Figure 3). Given the critical importance of this region of the kinases, modifications would be expected to be extremely rare. Indeed, this motif is highly resistant to alteration, as a broad assortment of kinases in the superfamily, despite large changes in substrate and supporting structures, have carefully retained it (Figures 1 and 2). AFK does contain an insertion between D166 and N171, demonstrating that such insertions can occur. However, the insertion in AFK changes the orientation of these residues very little, indicating that in this one case the insertion was acceptable precisely because it did *not* change the essential structure of the catalytic loop. However, ChaK and PIPKIIβ lack this element, instead using an approximately linear chain structure (with compensation in ChaK for the loss of N171, and no obvious compensation in PIPKIIβ; Figures 2 and 3). Thus, it seems reasonable that AFK and PI3K should be grouped relatively closely to the TPK/APH/CK cluster, despite more extensive structural divergence between these structures.

Though AFK is a protein kinase, and can be linked to the TPK/APH/CK cluster, it appears to be more closely related to PI3K than to the TPKs. Though the structural evidence for this linkage is weaker than that linking together the TPK/APH/CK cluster, it remains persuasive. First, though PI3K and AFK share a similar crossing loop structure to that seen in the TPK/APH/CK cluster, the specific residue motifs are changed. Instead of using a histidine or tyrosine residue at Y164 to form a hydrogen bond with the backbone of T183 in the other loop, AFK and PI3K both use an arginine residue at L167 to form this interaction (Figure 5). This interaction is shared by only these two structures. In addition, AFK and PI3K do not conserve an aspartate residue at D220 (seen in all other kinases containing the 3–10 helix motif in their catalytic loop) and the larger network of interactions that are seen in conjunction with this residue (Figure 5).

If structures outside of the conserved core are considered, AFK and PI3K have three similar helices in their C-terminal subdomains, one of which is highly superposable. The other two are weakly superposable, but not seen in any other structures in the superfamily (Figure 2). The net effect of the overall structure of both AFK and PI3K is that the enzyme is flat-faced [21,22]. As AFK is seen in only one species (Table 3), and PI3K is seen in many, a scenario in which PI3K and AFK evolved from a common ancestor might require that AFK evolve from a kinase similar to PI3K. Such a scenario is quite plausible, as even present-day PI3K has some protein kinase activity [65,66] (and enzymes can change their substrate specificity relatively easily over long evolutionary timescales [67]). In addition, a small family of Ser/Thr protein kinases has been identified that contain a catalytic domain highly similar to that seen in PI3K. These phosphoinositide 3-kinase related kinases (PIKKs) demonstrate that the PI3K catalytic domain can be readily modified to phosphorylate protein targets exclusively [68]. However, as with PI3K, these kinases do not share obvious sequence similarity with AFK. AFK may thus represent an alternate modification of a lipid kinase to become a pure protein kinase. Alternately, both AFK and PI3K may have independently converged upon the observed structural similarities as a result of the requirement to be flat-faced. However, our phylogenetic tree also shows AFK and PI3K to share a common ancestor, with relatively high posterior probability (Figure 4).

### PIPKIIβ and ChaK are Highly Divergent Kinase Structures, Both from the Rest of the Superfamily and from Each Other

Though ChaK and PIPKIIβ can be distinguished from other kinases in the superfamily based on their lack of a 3–10 helix in their catalytic loops, this does not mean they have any clear similarity to each other that would suggest a close evolutionary link. Indeed, these two kinases do not share any distinctive structure or sequence motifs, and appear no more similar to each other than to the 3–10 helix containing group. RMSD values and number of aligned positions between the two structures are no better than those for comparison of ChaK and PIPKIIβ with the rest of the superfamily (Table 4). Both kinases share an approximately linear catalytic region, but the way in which this structure is achieved is quite different. ChaK has short strands 13 and 14 (Strands 7 and 8), coupled to a novel structure of strands 12 and 15 (Strands 6 and 9) that avoids the use of a crossing loops in the C-terminal subdomain. PIPKIIβ uses elongated strands 10 and 12 (Strands 7 and 8), lacks Strands 6 and 9, and has crossing loops (Figure 2).

Though SCOP does not place PIPKIIβ in the same superfamily as the other kinases, a comparative study has linked this structure to the protein kinase–like superfamily [34]. Our analysis does not suggest any reason to doubt this linkage, but it does indicate that PIPKIIβ is the most divergent kinase in our set. For example, PIPKIIβ displays substantial changes in ion pair patterns and orientation of secondary structural elements (see analysis above and Table 2).

Since ChaK and PIPKIIβ are highly dissimilar, it follows that that they should not be considered close relatives. Both ChaK and PIPKIIβ have been suggested to provide possible links between the protein kinase–like superfamily and two other superfamilies containing mostly metabolic enzymes: the SAICAR synthase and ATP-grasp superfamilies [20,34]. In the case of PIPKIIβ, our analysis does not contradict this possibility. PIPKIIβ is extremely structurally distant from the rest of the superfamily (Table 4), and conserves only the most minimal set of residues related to ATP binding and catalysis, as well as a few hydrophobic residues that form shared hydrophobic cores (Figure 3). We attempted to place PIPKIIβ on our phylogenetic tree, both in an effort to illuminate its origins, and provide a possible outgroup for the tree. Remarkably, the tree places the origin of the PIPKs in the middle of the AKs. This region could be a likely “origin” point for the kinases, where an ancestral kinase diverged to form the AKs, as well as the TPKs (Figure 4). Thus, the phylogenetic tree results are consistent with a very distant relationship between PIPKs and the rest of the kinase superfamily. However, given the weak structural evidence for the location of PIPKs on the tree, this link should be considered speculative (while PIPKIIβ has many distinct structural features, most do not provide informative characters in our matrix for purposes of placing branches). Consideration of species distribution of the PIPKs indicates that they appear to be restricted to the eukaryotes (Table 3). This observation suggests that PIPKs are a more recent arrival into the arsenal of kinases, perhaps developed by eukaryotes in response to a heightened requirement for more complex signaling networks. However, if the PIPKs are a relatively recent invention, this precludes a role for them as a direct link between the SAICAR synthase and/or ATP-grasp folds and protein kinase–like superfamily. However, it does not preclude the possibility that the PIPKs and the kinase superfamily share a very distant common ancestor (which was not necessarily functionally a kinase). The PIPKs share notable structural similarity with the SAICAR synthetase family, leading them to be grouped within this superfamily in the SCOP database [1]. We speculate that the PIPKs may have become kinases through derivation from an ancient non-kinase fold, perhaps a protein similar to SAICAR synthetase. Hence, they may have become kinases through a process of “convergent divergence” with the rest of the kinase superfamily. In such a scenario, the PIPKs would have converged upon the same kinase activity that had already been discovered much earlier by their distant relatives in the rest of the kinase superfamily.

Though ChaK has also been suggested as a possible link between the kinase superfamily and the ATP-grasp superfamily [20], our results, as well as the work of others [27], cast considerable doubt upon this hypothesis. Consideration of the species distribution of α-kinases indicates that they are only narrowly distributed in eukaryotes, appearing primarily in metazoans, and completely absent from green plants (Table 3). This data suggests that the α-kinases appeared relatively recently in evolution, and thus they are precluded from being a direct link between two ancient and widely distributed superfamilies. Presumably, the α-kinases were derived from an extant kinase. However, determining the closest relative to the α-kinases is difficult because of the extremely divergent sequence and structure of ChaK.

Our Bayesian tree places ChaK well within the AKs, closest to PI3K and AFK. Though the posterior probability is relatively low for the branch separating these three families, it is high for the branch separating the three families and the PIPKs from the rest of the superfamily (Figure 4). This would suggest that the closest known structural relative to the α-kinases may be the PI3K family (since AFK apparently evolved recently and is narrowly distributed, it is precluded as a possible source protein for the derivation of the α-kinases). PI3K and ChaK do share a distinctive straightened Strand 4 (strand 6 in PI3K; strand 9 in ChaK, Table 2), but otherwise they do not have any clear structural similarity that would argue for a link. RMSD values for superpositions between these two proteins are unremarkable relative to the rest of the superfamily (Table 4).

Our conventional tree provides a completely contradictory scenario, but there are reasons to consider it as another plausible possibility. Not only does ChaK appear to radiate from the TPKs, it appears to radiate specifically from the AGC group, with rapid mutational events placing it at a great eventual distance from this group (Figure 6). Though bootstrap support for this origin for ChaK is weak, it is surprisingly strong compared with many other branches, especially given the extreme rearrangements in this structure. Remarkably, searches against the PDB with combinatorial extension (CE) [69] reveal that the strongest structural matches to ChaK are several PKA structures, members of the AGC group of TPKs (strongest match: PDB ID: 1CDK [70], CE Z-score = 4.1, CE RMSD = 4.1Å). By contrast, PI3K does not display such close structural similarity to ChaK (CE Z-score = 3.5, CE RMSD = 4.6Å). Further supporting an AGC group origin for ChaK is the presence of α-Helix B, a structure that is a distinctive feature of the AGC kinases (Figures 1–3 and Table 2).

We speculate that the α-kinases were developed to provide a novel signaling capacity useful to more complex eukaryotic organisms. Given the rapid divergence of the α-kinase family from the rest of the kinase superfamily, and the high level of sequence similarity within the α-kinase family [27], we suggest that the most likely scenario for the creation of the α-kinase family is a single catastrophic genetic event. This event could have perhaps taken the form of deletion of much of the C-terminal end of an extant kinase gene, or fusion of a kinase gene with another gene. While such an event would usually not lead to a functional kinase, this mutation would have produced a kinase that had the novel capability to phosphorylate α-helices.

If the α-kinases were derived from a TPK, it is possible that they contain a zinc finger because this was the way that a functional fold was “rescued” after severe modification of the c-terminal subdomain. It is intriguing that the zinc coordination site in the α-kinases is partly formed by a histidine residue, H1751(F154) in helix D (Helix E) of ChaK. Though H1751 does not structurally align with the conserved H158 seen in the AGC kinases (it is one turn up the helix from H158; Figure 3), it is possible that the presence of a highly conserved histidine in this region of the structure provided part of the initial zinc coordination site in the first α-kinase. Afterward, the location of the helix may have shifted in the α-kinase structure, or the histidine could have been replaced in a point mutation by H1751. Apparently, the first α-kinase underwent a period of rapid sequence change, perhaps to optimize its stability and function. Regardless of the source protein, this process would have led to its distinctive structure and great sequence distance from the TPKs and other AKs (Figures 2, 4, and 6)

### Conclusion

The kinase superfamily provides an interesting example of the types of changes seen in proteins over long evolutionary timescales. Lesk and Chothia were the first to perform an in-depth study of protein structure evolution [71]. They described a gradual evolutionary drift of sequence and structure in the globins, but with careful maintenance of the heme binding pocket essential to function.

The changes seen in the kinases are more severe at both the structure and sequence level. It would appear that a major driving force for these large structural changes is the diversity of substrates that kinases from the superfamily must recognize and phosphorylate. Kinase superfamily members phosphorylate an amazing array of targets, from small molecules such as choline (CK) [23], to loop-type regions of proteins (the TPKs) [29], to α-helices (α-kinases) [28], to membrane-bound phosphoinositides (the lipid kinases) [19,21]. The structural changes between families, particularly in the C-terminal subunit, allow for such interactions to take place. In other cases, structural changes have allowed the kinases in the superfamily to partner with accessory domains important to activity and/or regulation (e.g., [21]).

The kinases have been adapted for so many purposes that, in the end, all they have in common is the essential kinase function, and the fold required to carry it out. The large structural shifts seen outside of this region have obliterated sequence similarity outside of the universal core. Even within the core, notable structure and sequence changes have occurred, considering the direct role of this region in the essential function of these enzymes. However, where changes occur to the core that would affect function of the enzyme, there is generally clear compensation for the lost structures and residues, such that function is retained. This sort of plasticity has been previously noted in larger-scale studies of protein evolution [67,72]. The net effect of these sorts of changes is a very low degree of sequence similarity at the superfamily level, even within the core. With such weak sequence similarity between superfamily members, it will not be surprising if other proteins join the superfamily once their structures are solved. A number of divergent kinases have already been identified for which structures are not yet available [35,36].

In this study, we have sought to provide a framework for understanding the development of the kinase superfamily from a common ancestor. By incorporating structural information into our phylogenetic analysis, we have been able to provide a coherent scenario for the evolution of the kinases, with strong support for most of our predictions. Though some areas of kinase structural evolution are still in doubt, we believe the framework provided here will be valuable as structures for more members of the superfamily become available. We expect that many of these structures will be able to provide additional insights into the structural evolution of this rich and expanding superfamily.

## Materials and Methods

### Construction of the representative set of kinase structures.

We utilized the classification scheme provided by the SCOP [73] and ASTRAL [74] resources (version 1.65) as a guideline for structure selection. To produce a representative set from the SCOP/ASTRAL domains, the sequences for all structures in superfamily d.144.1 (“protein kinase–like”) were clustered via the single-linkage method using BLASTCLUST [75], such that no structure in any cluster could be aligned to a structure in any other cluster with sequence identity ≥ 45%. A single structure was then chosen from each cluster as the structural representative for that group. The choice of a 45% identity cutoff was based on the observation that sequences can be aligned with high accuracy above ~40% identity based on sequence information alone [41,42,76]. Hence, alignments between representative structures from each cluster were likely to benefit from the use of structural information, while structure-based alignment *within* a cluster would be unlikely to surpass the accuracy achievable with standard sequence alignment techniques. In addition, this filtration ensured that all structures included in the alignment would be evolutionarily divergent, and thus provide interesting information about structural and sequence conservation in the superfamily.

Representative structures were manually selected from each sequence cluster based on the following cascading tests: (1) Structures were favored if they were bound to ATP or an ATP analog, or if (for TPKs) they were in a “closed” conformation [14,16]. Structures bound to ATP (or “closed”) were more informative because their ATP interactions could be studied, they tended to have fully resolved loop regions, and they were easier to align and compare. (2) Higher-resolution structures were favored. (3) Structures with wild-type sequences were favored over structures with experimental sequence mutations.

As discussed in the Introduction, the structure of PIPKIIβ was also added to the set of structures, even though it is not a member of the same SCOP fold group as the other kinases (d.143.1, as opposed to d.144.1). New kinases are constantly being added to the PDB; this representative set was kept unchanged for the duration of the study to maintain the tractability of the dataset.

### Structural alignment of kinase representatives.

The representative kinase structures were first aligned using a variant of the CE method [69] modified to provide progressive multiple alignments of protein structures. Using this alignment as a starting point, the alignment was then completely overhauled manually, starting at the N-termini of the proteins and following the structural trace through to the C-termini. No regions were ignored or skipped (i.e., even loops were carefully considered and aligned). The alignment was constructed with the primary aim of maximizing the aligned positions between structures, provided that there was a rational basis for the alignment. This meant, for example, that secondary structural elements could be aligned even if they diverged spatially upon rigid body superposition. We also sometimes used transitive alignments to align portions of structures. This meant that when two elements were distant spatially between a pair of structures, a third structure was considered that provided a “bridge” between the first and second structures. The element could be aligned to the bridge structure for both structures, providing a rational alignment between an otherwise difficult-to-align structural pair. At all times, the alignment was guided by direct visual inspection of the structures, using the CE alignment viewing software [77] and other structure viewers as appropriate. In addition, sequence and structure alignments previously published by kinase experts were used as a guideline [13,17]. Finally, many of the initial publications reporting the structures in the representative set provided alignments to other kinases (see Table 1 for citations). These alignments were also considered where appropriate. Structures were aligned with the goal of providing an optimal alignment between each structure and all other structures in the set, as opposed to one or two other structures (e.g., the closest relative of the structure in question). This process was painstaking, but yielded an extremely high-quality alignment of the protein kinase–like superfamily that considered both structural and functional features. It should be noted that aligning structures with the goal of creating an optimal *multiple* alignment will, in many cases, produce slightly suboptimal alignments between any given pair of structures (this occurs because often there must be a “compromise” when pairwise alignments of shared structures are not consistent with each other). In practice, this is an issue only in ambiguous regions; the key highly conserved regions can be aligned optimally throughout the superfamily. However, our bias toward maximal alignment of positions and the issue of pairwise suboptimality resulted in relatively high RMSD values (Table 4). Alignments of equivalent segments with an automated method such as CE will often produce lower RMSD, but with fewer aligned positions. However, automated methods such as CE must limit their alignments of ambiguous regions to avoid alignment errors. When creating manual alignments, this limitation is removed. We believe the alignment to be of sufficient quality to serve as a “gold standard” for studying the kinases (and for benchmarking protein structure alignment methods as well). The alignment is available in several formats for download from http://www.sdsc.edu/pb/kinases.

### Analysis of the structure and sequence alignment.

The resulting residue equivalences from the manual alignment were used to produce both superpositions of the kinase structures and a corresponding sequence alignment. The sequence alignment was annotated and analyzed using the JOY software [78], which maps structural features onto sequence alignments. In order to standardize the classification of secondary structures, the DSSP [79] method as implemented in sstruc [80] in the JOY software was used as the final arbiter of secondary structure classification (Figure 3).

Analysis of residue conservation was achieved initially by careful visual inspection of the alignment. Conservation at sequence positions within each family was confirmed through the use of Consurf-HSSP [81] conservation data provided through the PDBsum database [82]. Further confirmation as to specific aspects of residue conservation (i.e., conservation of a specific residue to identity, or conservation of a specific property) was accomplished through survey of the family alignments provided in the Pfam database (where available) [56].

Analysis of the structures was performed with molecular viewing software, augmented with the JOY annotation results. The Chimera software [83] was used to create superpositions of structures based on the manual alignment (Table 4). Residues of particular interest were evaluated for hydrogen-bond interactions and other contacts via the CSU server [84].

### Phylogenetic tree construction.

The structure-based sequence alignment presented in Figure 3 was used as the basis of all sequence-based portions of the phylogenetic analysis (one TPK structure, Pak1 [85], has a non-wild-type K299R(K72R) substitution, which was reverted to a Lys in our sequence alignment when performing phylogenetic analysis). The tree presented in Figure 4 was constructed using Bayesian phylogenic inference in the program MrBayes [47]. A combined analysis was performed, using both the sequence alignment and the structural characters matrix in Table 2 as “mixed” data [38]. Structural characters were submitted to MrBayes as morphological (“standard”) characters. The characters were modeled as unordered (e.g., a character could change directly from 0 to 2 without having to pass through 1). Both the sequence data and morphology data were modeled with an independent gamma distribution of substitution rates, using the default approximation of four rate classes for each. MrBayes offers a wide selection of model priors for amino acid substitution, and ideally the best-fitting priors should be chosen for the final analysis. Preliminary runs with MrBayes using a mixture of model priors (using the option aamodelprior = mixed in the command prset) demonstrated conclusively that priors based on the substitution rates from the BLOSUM matrices [86] provided the best fit to the sequence alignment data (they had, by far, the highest posterior probability in the analysis). Therefore, the BLOSUM model was used to provide substitution priors for the amino acid sequence portion of the data. Morphological characters were modeled using the default substitution prior for “standard” characters provided in MrBayes. All other settings used in MrBayes were the defaults for the software. The simulation was run for 2,000,000 generations, with tree sampling every 100 generations, for a total of 20,000 trees. At the completion of the run, the “average standard deviation of split frequencies” (a metric in MrBayes to determine convergence of the simulation) was ~0.0084, well below the recommended maximum of 0.1 (MrBayes documentation). A tree was generated using the default methodology and the recommended “burnin” (discarding) of the first 25% of samples (i.e., the tree was generated using the final 15,000 of 20,000 samples). A file containing the input alignment, run settings, and instructions for replication of the MrBayes results is available at http://www.sdsc.edu/pb/kinases.

In order to ascertain the influence of the morphology and sequence datasets on the resulting mixed tree, similar runs were made with MrBayes on the sequence and morphology datasets alone. These runs used identical parameter settings to those for the mixed model for the corresponding datasets (except that they were run for a smaller number of generations). The sequence-only tree was run for 300,000 generations, after which the standard deviation of split frequencies was ~0.037. The structural characters-only tree was run for 500,000 generations, after which the standard deviation of split frequencies was ~0.011. Both runs were processed using the same procedures as above. The resulting trees are provided in Figures S1 and S2, and demonstrate that each of the two methods alone was unable to produce a resolved tree.

Trees produced in PHYLIP [87] used only the sequence alignment data (derived from the structure alignment), and did not consider the structural characters. The alignment was first subjected to bootstrapping via the SEQBOOT program (with default settings), producing 1,000 replicates. Sequence distances were then estimated for each replicate in the program PROTDIST. Since tests with MrBayes indicated that the BLOSUM-based model provided the best fit to the alignment data, distances between sequences were estimated using the PMB model of residue substitution, which is based on the BLOSUM matrices [88]. Substitution rates were modeled as following a gamma distribution, with α = 2.15 (the correct value for α was estimated using a preliminary run of MrBayes with the BLOSUM priors). Trees were constructed for each bootstrap replicate using the Fitch-Margoliash method [46] in the program FITCH. Finally, a single consensus tree was built from the resulting trees in the program CONSENSE, using the default “majority rule (extended)” mode (this method places branches in the final tree when they are seen in > 50% of the input trees; it then places branches with lower representation if they are consistent with the current branches, using cascading selection for highest bootstrap values). Branch lengths were estimated for the resulting tree using the original alignment to determine distances in PROTDIST. These branch lengths were then applied to the consensus tree using FITCH. A copy of the input alignment and instructions for replication of the results is available at http://www.sdsc.edu/pb/kinases.

## Supporting Information

Figure S1 Phylogenetic Tree Made with MrBayes, Using Only the Structure-Based Sequence Alignment in Figure 3 as Input Structures are labeled using a pseudo-ASTRAL ID code, in which positions 2–5 provide the PDB ID code, and the last position provides the specific chain from the PDB file (if applicable). Posterior probabilities are provided to the right of each resolved branch. Numerous polytomies are visible as horizontal branches that are not subdivided by internal branches. Where branches are resolved, posterior probabilities are usually lower than those for the tree in Figure 4. This figure and Figure S2 were created using TreeView [89].
(50 KB TIF)

Figure S2 Phylogenetic Tree Made with MrBayes, Using Only the Structural Characters Provided in Table 2
Structures are labeled using a pseudo-ASTRAL ID code, in which positions 2–5 provide the PDB ID code, and the last position provides the specific chain from the PDB file (if applicable). Posterior probabilities are provided to the right of each resolved branch. Numerous polytomies are visible as horizontal branches that are not subdivided by internal branches. Though the structural characters provided key information that significantly improved the tree in Figure 4, they are inadequate to discern relationships by themselves, particularly for the TPKs.
(40 KB TIF)

### Accession Numbers

The Protein Data Bank (http://www.rcsb.org/pdb/) accession numbers for proteins discussed in this paper are AFK (1CJA), APH(3′)-IIIa, (1J7U), ChaK (1IA9), CKA-2 (1NW1), Pak1 (1F3M), PI3K (1E8X), PIPKIIβ (1BO1), and PKA (1CDK).

## Abbreviations

AFK: actin-fragmin kinase
AK: atypical kinase
ChaK: channel kinase
Chk1: cell cycle checkpoint kinase
CK: choline kinase
CKA-2: choline kinase isoform A-2
PDB: Protein Data Bank
PI: phosphatidylinositol
PI3K: phosphoinositide 3-kinase
PIPKIIβ: type IIβ phosphatidylinositol phosphate kinase
PKA: protein kinase A
RMSD: root mean square deviation
SCOP: Structural Classification Of Proteins
TKL: tyrosine kinase–like
TPK: typical protein kinase
